# Case Report: Diagnosis of COVID-19 versus Tropical Diseases in Pakistan

**DOI:** 10.4269/ajtmh.20-0356

**Published:** 2020-05-05

**Authors:** Syed Muhammad Mashhood Ali Bokhari, Fatima Mahmood, Syed Muhammad Saud Ali Bokhari

**Affiliations:** 1 Nishtar Hospital, Multan, Pakistan;; 2 Shifa College of Medicine, Islamabad, Pakistan

## Abstract

A 25-year-old medical student presented in Multan, Pakistan with a high fever, cough, myalgia, and diarrhea consistent with the typical signs and symptoms of novel coronavirus disease (COVID-19). The patient had traveled to high COVID-19–risk areas within Pakistan and had no significant medical and surgical history. Based on nasopharyngeal and oropharyngeal swab testing, the patient was found to be negative for COVID-19. He subsequently developed a diffuse rash and had serology consistent with dengue and measles. The patient was treated symptomatically, and his condition gradually improved over 7 days. This case highlights the high prevalence of many tropical diseases in low-income countries and the need for clinicians to consider alternate diagnoses in addition to testing for COVID-19 during the pandemic.

## CASE

A 25-year-old man presented to the emergency department of Nishtar Hospital, Multan, Pakistan with a 3-day history of high fever, chills, dry cough, myalgias, and diarrhea. He reported a sudden onset of symptoms. The patient is a medical student with no prior medical history. He reported recent travel with his family to Islamabad and Karachi, regions with known novel coronavirus disease (COVID-19) transmission, but family members denied similar complaints. The patient’s childhood and adult immunization records showed that all immunizations were up to date.

Considering the emergence of COVID-19 and the patient’s travel history, he was admitted to the hospital. Droplet and contact precautions were initiated. The staff caring for the patient were provided appropriate personal protective equipment.

In the ward, the patient was continuously shivering, with a temperature of 39.5°C, pulse 140 per minute, blood pressure 95/60 mm Hg, respiratory rate 18 per minute, and oxygen saturation 99% while breathing ambient air. Breath sounds were equal and clear in both lung fields. Chest radiographs showed no abnormalities.

Initial laboratory tests were mostly unremarkable but showed mild elevation of liver function tests (Table 1). Nasopharyngeal and oropharyngeal swabs were tested for severe acute respiratory syndrome-coronavirus-2 by reverse transcriptase–Polymerase chain reaction, and both tests were negative. Therefore, we decided to steer the patient’s management toward other infectious diseases within our differential diagnosis.

**Table 1:** Clinical laboratory results

Variable	Reference range	Hospital day 1	Hospital day 3
Sodium (mmol/L)	136–145	137	138
Potassium (mmol/L)	3.5–5.1	3.2	3.3
Chloride (mmol/L)	98–107	101	100
Creatinine (mg/dL)	0.7–1.3	0.73	–
ESR (mm/hour)	1–20	28	–
Total protein (g/dL)	6.0–8.5	6.1	–
Albumin (g/dL)	3.2–4.8	3.3	–
Aspartate aminotransferase (U/L)	< 35	83	–
Alanine aminotransferase (U/L)	< 45	95	–
Alkaline phosphatase (U/L)	50–116	112	–
Lactate dehydrogenase (U/L)	120–246	240	–
Total bilirubin (mg/dL)	0.2–1.1	0.2	–
Hb (g/dL)	13–18	14.4	14.2
Hct (%)	38–52	42	44
Platelets (×10^9^/L)	150–400	157	145
White blood cell count (×10^9^/L)	4–11	7.4	8
Neutrophils (%)	40–75	50	52
Lymphocytes (%)	20–50	35	34
Monocytes (%)	2–10	10	8
Eosinophils (%)	1–6	5	6
INR	0.8–1.1	1.0	–

ESR = erythrocyte sedimentation rate; INR = international normalized ratio.

The patient was treated with intravenous hydration, ceftriaxone, metronidazole, and paracetamol. Initially, he experienced relapsing high fevers, productive cough, severe myalgias, and worsening diarrhea. On the third day of hospital admission, the patient developed a maculopapular rash on the face and trunk, which gradually spread to the extremities over 2 days. To evaluate the rash, measles and varicella antibody tests were also conducted.

Microscopy of thick and thin smears for malaria, serology and surface antigen markers for viral hepatitis, and bacterial blood and stool cultures were negative. Testing for dengue showed positive IgM titers (1.6 U/L) and borderline NS1 antigen (0.8 µg/mL) results. Further serologic investigation revealed elevated IgM (137 U/L) against measles virus and negative serology for varicella. The same treatment measures were continued. By day 7, the patient’s fever, rash, and diarrhea had subsided; intravenous access was removed; and he was discharged.

## DISCUSSION

The WHO defines tropical diseases as infectious diseases that thrive in hot and humid tropical conditions, including dengue, malaria, leishmaniasis, schistosomiasis, and onchocerciasis, among several others.^1^ Despite medical advancements and numerous control measures, many developing nations still face the burden of tropical diseases. Dengue fever is one of the most prevalent diseases in Pakistan, with more than 50,000 cases and resulting in more than 90 deaths in 2019.^2^ Multan district faced a devastating outbreak of dengue fever in 2015.^3^

In our case, the patient raised suspicion for concomitant measles infection based on the WHO clinical case definition, which defines suspected measles as fever and maculopapular rash with cough, coryza, or conjunctivitis.^4^ Although the appearance of high titers of measles IgM seems puzzling in an immunocompetent, immunized patient, possible explanations for infection could be waning immunity, secondary vaccine failure, or prolonged exposure to a measles patient.^5^ Aggressive vaccination campaigns against measles were accompanied by a 71% reduction in mortality and morbidity rates worldwide between 2000 and 2011, but Pakistan continues to face this disease burden, with 94 measles deaths in Punjab during early 2013.^6^

Although COVID-19 is emerging as a pressing threat worldwide, this case underscores the need for clinicians practicing in Pakistan and other tropical regions to consider the possibility of common tropical diseases in all suspected patients. It also highlights the challenges facing the measles elimination campaign and the common threat of dengue in Pakistan.

## Figures and Tables

**Table 2:** Microbiology report

Test	Reference range	Results
Novel coronavirus disease RT-PCR test	–	Negative
Dengue virus IgM (U/L)	< 0.9	1.59
Dengue NS1 antigen (µg/mL)	< 0.6	0.8
Measles IgM (U/mL)	< 20	136.6
Influenza	–	Negative
Stool culture	–	No growth after 7 days
Blood culture	–	No growth after 7 days
Thick and thin smear microscopy for malaria	–	Negative

RT-PCR = reverse transcription polymerase chain reaction.

## References

[1] Ross R, 1901 Tropical diseases. J Am Med Assoc XXXVII: 450–451.

[2] World Health Organization, 2019 Epidemic and pandemic-prone diseases Outbreak update – Dengue in Pakistan, 1 December 2019 [Internet]. Available at: http://www.emro.who.int/pandemic-epidemic-diseases/dengue/outbreak-update-dengue-in-pakistan-1-december-2019.html.

[3] Saba S Khan AUR Naeem-Ullah U Bokhari SHM, 2019 Clinical profiles of dengue fever patients, during an outbreak. J Arthropod Borne Dis 13: 126–134. 31803773 PMC6885141

[4] Dietz FJ Nieburg P Gubler DJ Gomez I, 1992 Diagnosis of measles by clinical case definition in dengue-endemic areas: implications for measles surveillance and control. Bull World Health Organ 70: 745–750. 1486671 PMC2393413

[5] Hickman CJ 2011 Laboratory characterization of measles virus infection in previously vaccinated and unvaccinated individuals. J Infect Dis 204 (Suppl 1): 549–558. 10.1093/infdis/jir106 21666212

[6] Khan T Qazi J, 2014 Measles outbreaks in Pakistan: causes of the tragedy and future implications. Epidemiol Rep 2: 1.

